# Multiple endocrine neoplasia type 1 with Zollinger–Ellison syndrome: clinicopathological analysis of a Japanese family with focus on menin immunohistochemistry

**DOI:** 10.3389/fendo.2023.1221514

**Published:** 2023-10-06

**Authors:** Noriko Kimura, Yasuji Hirata, Nozomu Iwashiro, Hiroshi Kijima, Shinobu Takayasu, Satoshi Yamagata, Satoru Sakihara, Shinya Uchino, Masanori Ohara

**Affiliations:** ^1^ Department of Clinical Research, National Hospital Organization Hakodate Hospital, Hakodate, Hokkaido, Japan; ^2^ Department of Diagnostic Pathology, National Hospital Organization Hakodate Hospital, Hakodate, Hokkaido, Japan; ^3^ Department of Hematology and Oncology, National Hospital Organization Hakodate Hospital, Hakodate, Hokkaido, Japan; ^4^ Department of Surgery, National Hospital Organization Hakodate Hospital, Hakodate, Hokkaido, Japan; ^5^ Department of Pathology, Hirosaki University Graduate School of Medicine, Hirosaki, Aomori, Japan; ^6^ Department of Endocrinology and Metabolism, Hirosaki University Graduate School of Medicine and Hospital, Hirosaki, Aomori, Japan; ^7^ Institute of Human Nutrition, Columbia University Irving Medical Center, New York, NY, United States; ^8^ Division of Diabetes and Endocrinology, Aomori Rosai Hospital, Aomori, Japan; ^9^ Department of Endocrine Surgery, Noguchi Thyroid Clinic and Hospital Foundation, Beppu, Oita, Japan

**Keywords:** multiple endocrine neoplasia type 1, Zollinger-Ellison syndrome, neuroendocrine tumor, pulmonary atypical carcinoid, metastasis, prognosis, menin, immunohistochemistry

## Abstract

**Background:**

Multiple endocrine neoplasia type 1 (MEN1) is an autosomal dominant disorder characterized by the occurrence of multiple epithelial neuroendocrine tumors (NETs) and non-NETs in various organs. *MEN1* encodes a 610-amino acid-long tumor suppressor protein, menin. The optimal treatment for multiple tumors, identification of the most critical tumors for patient prognosis, and menin immunohistochemistry findings remain controversial. Therefore, we aimed to elucidate these issues through a histological analysis of tumors and tumor-like lesions in a Japanese family, comprising a father and his two sons, who had MEN1 with Zollinger–Ellison syndrome (ZES).

**Patients and methods:**

All family members had a germline alteration in exon 10, c.1714-1715 del TC of *MEN1*, and exhibited multiple synchronous and metachronous tumors. The patients had pulmonary NETs, hyperparathyroidism, hypergastrinemia, pituitary adenomas, pancreaticoduodenal NETs, adrenocortical adenoma with myelolipoma, nodular goiter of the thyroid, lipomas, and angiofibroma. Most tumors were resected and histologically examined. We compared their clinical courses and tumor histology, and conducted menin immunohistochemistry (IHC).

**Results:**

Two patients died of pulmonary NET G2. One patient who underwent pancreaticoduodenectomy was cured of ZES; however, the two other patients who did not undergo pancreaticoduodenectomy suffered persistent ZES despite treatment with octreotide. Menin IHC revealed varying NET intensities, ranging from positive to negative stains.

**Conclusion:**

Pancreaticoduodenectomy is the most effective treatment for ZES. Long-term follow-up is essential for pulmonary NET G2 owing to the risk of distant metastasis and/or multiplicity. Moreover, the variability of menin IHC in MEN1-related tumors may indicate the pattern of tumor formation rather than the diagnostic utility of menin in MEN1.

## Introduction

Multiple endocrine neoplasia type 1 (MEN1) is an autosomal dominant disorder with a high degree of penetrance characterized by the co-occurrence of multiple endocrine neoplasia/hyperplasia and non-endocrine neoplasia of the skin and soft tissues (1). *MEN1* is located on chromosome 11q13 and comprises 10 exons with over 450 germline mutations (1–4). It encodes a 610-amino acid-long tumor suppressor protein, menin. MEN1 is defined clinically by the occurrence of multiple epithelial neuroendocrine tumors (eNETs), such as parathyroid adenoma/hyperplasia (up to 100%), pancreaticoduodenal eNETs (30–90%), pituitary adenoma (30–40%), bronchopulmonary tract eNETs (3–10%), and tumors of the gastrointestinal tract such as gastrinoma (50–80%), as well as non-eNETs, such as adrenocortical adenoma/carcinoma (20–40%) and breast carcinoma (7%) (2–5). Concomitant diseases of the skin and soft tissue, such as angiofibroma, collagenoma, café-au-lait macule (40–80%), and multiple lipomas (10%), have been reported (2, 6). Multiple combined tumors and tumor-like lesions with metachronous appearance complicate MEN1 diagnosis and treatment. Zollinger–Ellison syndrome (ZES) caused by hypergastrinemia occurs in 30% of patients with MEN1 (7, 8).

This study assessed a Japanese family, comprising a father and his two sons, who had MEN1 with ZES. The family members exhibited a germline alteration in exon 10—c.1714-1715 del TC of *MEN1* (the reference number for the transcript and protein nomenclature were NM_130799 and p.Ser572Glufs*24, respectively). The gene mutation of Case 1 (proband) was reported by Hai N et al. as Case 16 in their article (9). They exhibited multiple tumors or tumor-like lesions, with the order of the tumor appearance varying between the patients. In this family, only one patient exhibited a good prognosis, with the two other patients dying of the disease. In this study, we discuss the most important factors for patient prognosis and treatment. Since systematic investigations on the immunohistochemistry (IHC) of menin in MEN1-associated tumors are currently limited (10, 11), it remains uncertain whether the immunohistochemical analysis of menin could assist in the diagnosis of MEN1-related tumors. Hence, we examined the IHC of menin in detail and discussed menin expression and tumor formation.

## Patients and methods

The proband of the family was a man in his late 20s (Case 1, Patient II-1) who has a father in his early 50s (Case 3, patient I-1) and a younger brother in his late 20s (Case 2, Patient II-2), all of whom were affected by various disease related with MEN1. The patients were definitely diagnosed with MEN1 through both clinical and genetic testing with their consent at their early 40s for Cases 1 and 2 and late 60s for Case 3. The family pedigree/genogram of the studied family is unknown except for that of the above members. The clinical summary and laboratory data of the patients are as follows:

### Case 1

The proband underwent surgery for an atypical carcinoid (AC) (eNET, G2) of the lung in the right lower lobe in his late 20s. Five years later, he underwent surgery again for the resection of an adrenocortical adenoma with myelolipoma in the right adrenal gland and an angiofibroma of the skin. Then, the following year, he was diagnosed with a pituitary adenoma with hyperprolactinemia and underwent surgery for the resection of a nodular goiter of the right thyroid gland and a lipoma in the right side of the neck. In his late 30s, he was found to exhibit multiple duodenal ulcers, hypersecretion of gastric juice, and hypergastrinemia. He was treated with octreotide, which significantly decreased gastrin plasma levels from 1300 to 353 pg/mL (normal range: 37–172 pg/mL). However, after several months, his plasma gastrin levels increased again despite treatment with octreotide. Several biopsies of the duodenal mucosa conducted by gastroduodenal endoscopy failed to show a gastrin-producing tumor at that time. Thereafter, his pancreatic body and tail were resected based on the suspicion of gastrinoma; however, microscopic examination revealed several microadenomas, including glucagon- and pancreatic polypeptide-producing adenomas, and the absence of gastrin-producing adenomas, in the pancreas. At approximately 40 years of age, he was found to exhibit urothelial lithiasis, hydronephrosis, hypercalcemia, and high parathyroid hormone (PTH) levels. In his early 50s, his intact PTH, prolactin, and gastrin plasma levels were 161 (normal range: 10–65), 79.4 (normal range: 3.6–12.8), and 1157 pg/mL, respectively.

**Figure 1:**
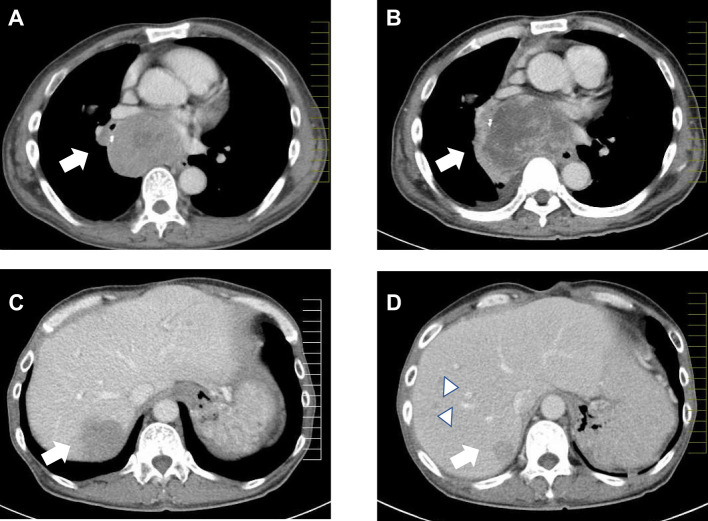
Computed tomography images of tumors in the mediastinum **(A**, **B)** and liver **(C**, **D)** of Patient II-1. The arrows indicate the main tumors. Comparing tumor size before the initiation of octreotide treatment and transcatheter arterial chemoembolization **(A)** and 10 months after treatment showed an enlargement of the posterior mediastinal tumor **(B)**. The liver tumor in segment S7 shrunk after treatment **(D)** when compared to its size 10 months before then **(C)**. Multiple new liver tumors were confirmed (**D**, arrowheads).

He was subsequently transferred to another hospital due to difficulty of clinical treatments and was comprehensively examined and retreated; in addition, he underwent a parathyroidectomy due to hyperparathyroidism. Tumors of the upper lobe of the right lung with multiple lymph node metastases in the right pulmonary hilar, upper posterior mediastinal, and around the pancreatic head regions were observed (**Figure 1**). Fluorodeoxyglucose positron emission tomography (FDG-PET) revealed similar lesions measuring 69 × 86 mm in his ninth rib and mediastinum. Using venous sampling following selective intra-arterial calcium injection, hotspots for gastrin were found in his duodenal mucosa. Although multiple liver tumors were confirmed in the patient by imaging, biopsies could not be performed owing to poor physical condition. However, venous sampling after calcium infusion confirmed that plasma gastrin levels were not elevated, suggesting that the liver tumors were likely not metastases of gastrin-producing tumors. Furthermore, gastrin-producing tumors were not detected by gastroduodenal biopsy. Consequently, the duodenal regions of the patient were not resected. Transcatheter arterial chemoembolization using mitomycin C and degradable starch microspheres was performed on the patient thrice in 10 months to treat multiple liver tumors. In the same year, he was diagnosed with a growth hormone- and prolactin-producing pituitary adenoma. Octreotide long-acting repeatable was administered to the patient to manage hypergastrinemia and elevated growth hormone levels, as well as to control NETs. An anti-prolactin secretion drug (cabergoline) was administered to him to treat hyperprolactinemia. Computed tomography images revealed that the liver tumor in segment S7 had shrunk 10 months following the first administration of octreotide and transcatheter arterial chemoembolization; however, the number of tumors in the liver increased. FDG-PET also revealed similar metastatic lesions in the posterior mediastinum, right lung, and right ninth rib, with elevated plasma serotonin levels (230 [normal range: 1.8–6.1] ng/mL), further confirming AC metastasis. The patient was subsequently monitored at home, but unexpectedly died in his early 50s due to an unidentified cause. An autopsy was not performed. The disease duration was 23 years.

### Case 2

For Patient II-2, urothelial lithiasis in the ureter and hyperparathyroidism were diagnosed in his early 30s. He underwent parathyroidectomy of two glands, the examination of which revealed chief cell hyperplasia; following this, hyperparathyroidism was controlled. The following year, he underwent surgery for the removal of a prolactinoma of the pituitary gland. He experienced ZES with duodenal ulcers and continued diarrhea in his late 30s. Although gastroduodenal endoscopy with biopsy did not reveal duodenal gastrinoma in the patient, he underwent pancreaticoduodenectomy two years later. He was cured of hypergastrinemia following pancreaticoduodenectomy. The patient developed neither pulmonary carcinoid nor tumor/tumorous lesions in the thyroid gland, adrenal gland, and soft tissues. Presently, he is alive and in his early 50s.

### Case 3

For this patient, multiple lipomas in his left shoulder and right thigh were resected in his late 60s. He was diagnosed with a prolactinoma of the pituitary gland and treated with cabergoline. Then, he was diagnosed with parathyroid hyperplasia and adrenocortical adenoma one year later. Two months after this, he was observed to exhibit gastrin hypersecretion and polyposis in the stomach and duodenum, for which gastroduodenal endoscopy with biopsy was subsequently performed. However, histological diagnosis revealed no gastroduodenal NET. He was diagnosed with ZES with hypergastrinemia in his early seventies. Surgeries for lesions in his pancreas and parathyroid glands were not performed because he did not consent. In the same year, he experienced acute myocardial infarction. Two years later, cytology confirmed the presence of an atypical carcinoid in the lower lobe of his right lung. Furthermore, in that year, he underwent surgery for a thoracic aortic aneurysm. He died of respiratory distress caused by the atypical carcinoid in the same year he underwent thoracic surgery. Autopsy was not performed. **Table 1** summarizes the tumor locations, tumor sizes, and histological findings of the patients (**Table 1**).

**Table 1:** Clinicopathological data of the MEN1-affected family with tumor location, size, and histology.

	Tumor location	Tumor size (mm)	Histology (mm)
**Case 1, Patient II-1** (proband)	Lung, right lower lobe (B9a)	18×10	NET G2, atypical carcinoid
	Adrenal cortex	50×35×20	Adrenocortical adenoma with myelolipoma
	Skin		Angiofibroma
	Pituitary gland	5.0, not operated	(hyperprolactinemia)
	Thyroid gland	18×15×12	Nodular goiter
	Neck	65×50×10	Lipoma
	ZES with multiple duodenal ulcers with hypergastrinemia		
	Pancreas (body & tail)	no macroscopic tumors	multiple micro-NETs, G1, 0.9–7.0, 2–4/glass slide
	Parathyroid gland (two)	14×7, 5×4	Hyperplasia vs Adenoma
	Lung, right upper lobe with multiple metastases to the lymph nodes, rib, mediastinum, and liver	35, cStage IV	NET G2 with coagulation necrosis
	Pituitary gland	8, not operated	(High growth hormone and prolactin levels)
	Died		
**Case 2, Patient II-2**	Parathyroid gland	left upper region: 17×11×8 right region: 8×4×5	Chief cell hyperplasia vs. adenoma Chief cell hyperplasia vs.adenoma
	Pituitary gland	3.0×3.0	prolactinoma
	Zollinger–Ellison syndrome		(hypergastrinemia)
	Duodenum, 2nd portion including a part of the lower bile duct	Multiple submucosal tumors	micro- NETs, G1, 1.2–8.0 micro- NETs in bile duct, G1, 1.3
	Lymph node	Locoregional, two, 12×5	Metastasis of the duodenal NET
	Pancreas	no macroscopic tumors	micro-NETs, G1, 2.0–8.0, two/glass slide
**Case 3, Patient I**	Shoulder and thigh	90×70, 110×80	Lipoma
	Pituitary gland	not operated	(hyperprolactinemia)
	Parathyroid gland	not operated	(hyperparathyroidism)
	Adrenal cortex		adrenocortical adenoma
	Pancreas	not operated	
	Zollinger–Ellison syndrome		(hypergastrinemia)
	Lung, right lower lobe	cT4N0M0	Atypical carcinoid (only cytology specimen)
	Died of respiratory failure		

### IHC study

Tumors were fixed in 10%-buffered formalin and embedded in paraffin. After reviewing all sections stained with hematoxylin–eosin, suitable sections were selected for IHC. IHC for gastrin, chromogranin A (CgA), synaptophysin, CD56, and Ki67 was routinely performed for all tumors of family members with MEN1. Moreover, we used antibodies to evaluate serotonin IHC, calcitonin to characterize lung tumors, insulin, glucagon, somatostatin, and pancreatic polypeptide to characterize pancreatic tumors, and CgA to differentiate parathyroid tumors from the thyroid gland. Menin expression was evaluated in tumors of the parathyroid gland, duodenum, pancreas, and lungs by IHC, which was performed on a VENTANA BenchMark ULTRA Slide Staining System (Roche Diagnostics, Indianapolis, IN, USA) using primary antibodies against CgA (DAKO; DAK-A3), synaptophysin (Nichirei, Tokyo, Japan; clone 27G12), CD56 (Nichirei clone MRQ42), Ki67 (MIB1) (Ventana; clone 30-9), somatostatin (Nichirei, polyclonal), gastrin (Dako, polyclonal), insulin (BioGenex, San Ramon, CA, USA, polyclonal), glucagon (Dako, polyclonal), and pancreatic polypeptide (Dako. polyclonal). Appropriate positive and negative controls were included in the experiment. Only menin (Santa Cruz Biotechnology, Inc.; Clone B-9, 1:200) was stained using the Histofine SAB-PO kit (Nichirei, Tokyo, Japan). Primary antibody against menin, which is mouse monoclonal antibody against amino acids 1–300 of the human menin protein, was incubated at 4°C overnight and secondary antibody was subsequently added at 18°C. We used placental and adult testis tissues as positive controls for menin (12, 13) and phosphate-buffered saline for negative stains.

To count menin-labelled cells, two of the most highly labelled areas (hot spots) were photographed at a magnification of ×400 and the cells were counted using a digital image analyzer (Lumina Vision, Mitani Corp, Tokyo, Japan). The procedure for counting menin-labelled cells was similar to that used for counting Ki67-labelled cells with the same instrument. Menin-positive and menin-negative cells (non-labelled cells) were counted and their ratio in each tumor determined. Normal pancreatic acinar cells were evaluated as positive controls (**Table 2**).

**Table 2:** Immunohistochemical study of the tumors of the MEN1 family.

Case (Patient)	Histology, Organ	Immunohistochemistry	
		General antibodies for NETs	Menin IHC, positive or negative (ratio of positive cells/total cells) Normal pancreatic acini (positive control) (3576/3964:90%)
Case 1 (Patient II-1)	NET G2 (Atypical carcinoid), right lower lobe, Lung	CgA +, serotonin +, CD56+, synaptophysin +, CK7+, CK20-, calcitonin -, gastrin -SSTR2A+, Ki67LI: 3%	Negative (136/1191: 11%)
	Adrenocortical adenoma with myelolipoma, Adrenal		Negative (24/2828: 0.01%)
	Angiofibroma, Skin		n.e.
	Adenomatous goiter, Thyroid		Positive
	Multiple micro-NETs, under 10 mm in size, Pancreas	gastrin -, CgA+, glucagon +, pancreatic polypeptide +, insulin -, somatostatin -	Negative (213/2453: 9%)
	Adenoma, Parathyroid	CgA+	Negative (512/4162: 12%),
	NET G2, right upper lobe, and posterior mediastinum, Lung	CgA+, CD56+, serotonin +, gastrin -, synaptophysin +, CK7+, CK20-, SSTR2A+, Ki67LI: 4%	Negative
Case 2 (Patient II-2)	Adenoma, Pituitary	prolactin +, growth hormone -	n.e.
	NETs, G1, Duodenum & lower Bile duct	gastrin +, CgA +, synaptophysin +, somatostatin +	Positive, or Mixed positive and negative cells (425/1208: 35%)
	Lymph node metastasis	gastrin +, CgA+	Mixed positive and negative cells
	Multiple micro-NETs, Pancreas	gastrin -, CgA+, glucagon +, pancreatic polypeptide +, insulin -, somatostatin -	Negative cells, or Mixed positive and negative cells
Case 3 (Patient I-1)	Atypical carcinoid, Lung	CgA +, serotonin+, gastrin -	n.e.

n.e., not examined; CgA, chromogranin A; CK, cytokeratin; SSTR2A, somatostatin receptor type 2A.

## Results

### Tumor histology with IHC

**Figure 2:**
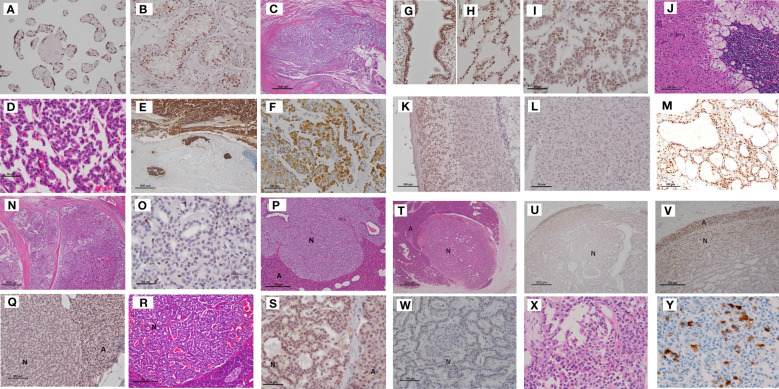
Tumors in Case 1 (proband), including bronchial NETs, the adrenocortical adenoma with myelolipoma, pancreatic NETs, and mediastinal NETs. **(A, B)** Positive controls for menin immunohistochemistry. The nuclei of chorionic villi and testicular tubular cells were positive for menin. **(C)** Bronchial NETs. Small tumor cells with hyperchromatic nuclei, forming a solid mass, extended into the bronchial lumen. Pulmonary tumor cells showed a trabecular arrangement. **(D)**. Tumor cells were strongly reactive to chromogranin A and invaded the lymph vessels **(E)**. In addition, they were positive for serotonin **(F)**. Normal bronchial and alveolar cells were positive for menin **(G, H)**. Bronchial NET cells demonstrated a mixture of positive and negative menin immunoreactivity **(I)**. (Scale bar: 1000 μm in **C**, 50 μm in **D**, 500 μm in **E, G**, 100 μm in **F**, and 50 μm in **H, I**). **(J–L)** Adrenocortical adenoma with myelolipoma. Monoclonal proliferation of oxyphilic adrenocortical cells combined with myelolipoma limbed by hyperplastic adrenocortical cells of the clear cell type **(J)**. Menin immunohistochemistry showed positive staining in residual adrenal cortical cells **(K)** and negative staining in adrenocortical tumor cells **(L)**. (Scale bar: 100 μm in **J, K**, and 50 μm in **L**). **(M)** Positive menin immunostaining in the nuclei of thyroid follicular cells of the adenomatous goiter. **(N)** Parathyroid adenoma separated with sclerosing stroma. Tumor cells comprised menin-negative and menin-positive cells **(O)**. (Scale bar: 500 μm in **N**, and 50 μm in **O**) **(P)** Microadenomas of the pancreas, the tumor cell nuclei of which were completely negative for menin **(Q)**. In contrast, another islet (HE staining) **(R)** showed positive menin staining **(S)**. A NET **(N)** measuring 7 mm in diameter was surrounded by acinus **(A)** (HE staining). **(T)** Showed a transition of menin immunoreactivity from the periphery to the center of the tumor nodule; the cells in the peripheral area of the NET showed positive but weaker staining for menin than acinar cells of the pancreas **(U)**. Higher magnification of figure **(U)** clearly demonstrated nuclear staining of menin in the peripheral area of the pancreas NET **(V)**. Higher magnification revealed that most tumor cells in the center were negative for menin **(W)**. (Scale bar: 100 μm in **Q**, 50 μm in **R** and **S**, 500 μm in **U**, 250 μm in **V**, and 100 μm in **W**.) **(X)** Mediastinal tumor comprising small tumor cells with a high nuclear–cytoplasmic ratio, which were focally positive for serotonin **(Y)**, and was diagnosed as an atypical carcinoid (NET G2). NET, neuroendocrine tumor; HE, hematoxylin and eosin.

NETs or tumor precursor lesions (hyperplasia or minimal NETs) in the family members exhibited a similar histological pattern, and comprised cells with hyperchromatic nuclei and eosinophilic cytoplasm with a high nuclear–cytoplasmic ratio, and were arranged in trabecular, insular, glandular, or solid patterns. The tumor cells were characterized based on their IHC reactivity to cytokeratin (AE1/AE3 or CAM 5.2) and neuroendocrine markers, including CgA, synaptophysin, and CD56. Menin expression was observed in the nuclei of the human placental and testis cells, which served as positive controls (**Figures 2A, B**).

### Tumors of case 1

The bronchial tumor of the patient comprised small cells arranged in a trabecular pattern, occupying the bronchial lumen with lymph vessel invasion. The tumor cells were strongly positive for CgA and synaptophysin, with a Ki67 labeling index of 3.0%. They were positive for serotonin but negative for calcitonin and gastrin. The tumor was diagnosed as AC (NET G2) of the bronchus. Menin IHC revealed nuclear staining in normal bronchial and alveolar cells. Tumor cells of the bronchial NET G2 displayed a mixture of menin-positive and menin-negative cells (**Figures 2C–I**).

The adrenocortical tumor of the patient, which measured 5.0 cm × 3.5 cm, comprised adrenocortical adenoma mixed with adipose tissue and bone marrow cells (myelolipoma). Menin expression was observed in the nuclei of normal adrenal cortex cells; however, the adrenocortical adenoma was negative for menin (**Figures 2J–L**).

The nodular goiter of the thyroid gland showed strong menin IHC in follicular cells (**Figure 2M**). The parathyroid tumor exhibited a nodular growth pattern with sclerosing stroma. The tumor cells were confirmed positive for CgA for differential diagnosis from thyroid nodules. The tumor demonstrated mixed menin-positive and menin-negative cells (**Figures 2N, O**).

Tumor lesions were not observed in gross pancreatic NETs; however, we identified 2–4 micro-NETs measuring 0.9–7.0 mm in diameter on each glass slide. These micro-NETs displayed varying degrees of menin immunostaining. For example, one NET with solid growth was completely negative for menin IHC, while the glandular-type NET exhibited positive staining for menin. Another pancreatic NET with a glandular structure showed gradation of menin IHC from the periphery to the center of the tumor nodule. Cells at the periphery of the NET were positive but weakly stained as compared to pancreatic acinar cells. Higher magnification revealed that most tumor cells in the center were negative for menin (**Figures 2P–W**).

The posterior mediastinal tumor was examined only by biopsy and was diagnosed as NET G2, and was positive for CgA and serotonin but negative for gastrin (**Figures 2X, Y**). Its Ki67 labeling index was 4%.

### Tumors of case 2

**Figure 3:**
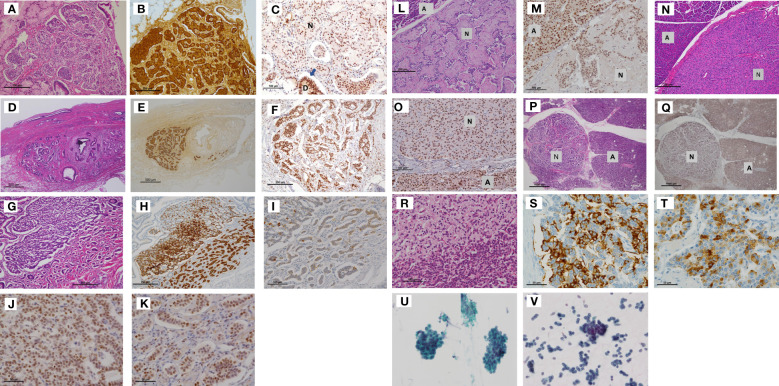
Tumors of Case 2 (including duodenal NETs, the lower bile duct NET, pancreatic NETs) and Case 3. **(A)** Submucosal NET in the duodenum measuring 1.0 mm × 1.0 mm, which demonstrated strong staining for gastrin **(B)**. Tumor cell nuclei were weakly positive for menin **(C)**. **(D)** Nest of small submucosal NETs (1.0 mm) in the bile duct. (Insert letter: D is residual duodenal gland, and N is NET). The cell nest expressed gastrin **(E)** and exhibited mixed strong and weak nuclear staining for menin **(F)**. (Scale bar: 250 μm in **A** and **B**, 100 μm in **C**, 500 μm in **D** and **E**, and 250 μm in **F**). The other duodenal NETs **(G)** were strongly positive for somatostatin **(H)** but very focally positive for gastrin **(I)**. Higher magnification demonstrated mixed expression of menin-positive and menin-negative nuclei **(J, K)**. (Scale bar: 250 μm in **G** and **H**, 100 μm in **I**, and 50 μm in **J**–and **K**.). A pancreatic NET with sclerosing stroma **(L)** and other small NETs of 2.0 mm in diameter **(N)** demonstrated weaker menin immunoreactivity than acinar cells **(M, O)**. The other micro-NETs **(P)** were almost negative for menin **(Q)**. Some NETs exhibited clusters of clear cells **(R)**. These micro-NETs showed foci of positive cells for glucagon **(S)** or pancreatic polypeptide **(T)** but were negative for insulin. (Insert letter: A is acini and N is NET. Scale bar: 250 μm in **L**, 100 μm in **M**, 250 μm in **N**, 100 μm in 3O, 1000 μm in 3P and 3Q, 100 μm in **R**, and 50 μm in **S** and **T**). The bronchial brushing cytology material of Case 3 **(U)** was similar to that of Case 1 (the proband) **(V)**, leading to a bronchial NET G2 diagnosis. NET, neuroendocrine tumor.

We identified micro-NETs measuring below 1.5 mm in diameter in the duodenum of the patient. These lesions were strongly positive for gastrin. Menin IHC revealed positive staining in the micro-NETs with lower intensity as compared to that in the adjacent duodenal mucosa. A micro-NET with a diameter of 0.7 mm was found in the lower bile duct of the patient and showed strong positivity for gastrin and menin. Another NET in the duodenum exhibited glandular and insular structures comprising cells positive for somatostatin, and showed only focal positivity for gastrin and a mixture of positive and negative cells for menin (**Figures 3A–K**).

In the pancreas of the patient, one to two micro-NETs measuring 2.0–8.0 mm in diameter were identified in each paraffin section. Varied menin immunostaining was observed in the pancreatic NETs. A NET with sclerosing stroma and other micro-NETs with a diameter of 2.0 mm exhibited positive but weaker menin staining as compared to acinar cells. The other micro-NETs were almost negative for menin. Some NETs exhibited clusters of clear cells and showed foci of positive cells for glucagon or pancreatic polypeptide (**Figures 3L–T**). Gastrin was totally negative in pancreatic NETs.

### Tumors of case 3

As the patient refused surgery for the tumors except lipomas, most were only diagnosed clinically using imaging and laboratory data. Only bronchial brushing was performed; the cytology specimen was examined and found to be similar to that of Case 1 (**Figures 3U, V**), leading to a bronchial NET G2 diagnosis.

### Menin-labelled cells in tumors

The ratio of menin-positive cells to menin-negative cells was found to be 90% for normal pancreatic acinar cells, 35% for duodenal NETs, 12% for parathyroid adenomas, 11% for pulmonary NETs, 9% for pancreatic NETs, and 0.01% for adrenocortical adenoma (myelolipoma).

### Brief summary of the cases

A Japanese family comprising a father and his two sons suffered from MEN1 with ZES, with a germline alteration in exon 10—c.1714-1715 del TC of *MEN1*. They had full sets of MEN1 tumors and tumor-like lesions. Only one patient who underwent pancreaticoduodenectomy survived, with the other two dying of the disease. However, it is unclear whether the cause of their death was related to ZES treatment. Menin immunohistochemistry and its significance in tumorigenesis and pathologic diagnosis is of significant research interest.

## Discussion

MEN1 is characterized by the development of multifocal small NETs and non-NETs in multiple organs. One clinically important difficulty is detecting the locations of gastrinomas, which induce hypergastrinemia in patients with ZES. In this study, we elucidated the characteristics of ZES and its possible treatments, the prognosis of patients with MEN1-associated bronchial NETs, and various menin IHC expression levels in MEN1-associated tumors.

### Zollinger–Ellison syndrome and treatments

We focused on the localization of gastrin-producing tumors in Cases 1 and 2. Conventional imaging techniques, such as CT and MRI, were used to localize non-functional/functional neuroendocrine neoplasms in each patient; however, no tumors were detected. MRI and CT are of limited use for the localization of gastrinomas as they are often small, multiple, and located in the duodenal submucosa (14). The patient in Case 1 experienced hypergastrinemia and duodenal ulcers for an extended period and underwent partial pancreatectomy. However, gastrin-producing cells were not detected in the multiple pancreatic NETs of the patient. In contrast, in Case 2, hypergastrinemia improved in the patient following pancreaticoduodenectomy, and histological examination revealed multiple micro-NETs in his duodenum and lower bile duct with lymph node metastases. No NETs in other locations had gastrin-positive cells. Duodenal micro-gastrinomas are the primary cause of ZES in most patients with MEN1, highlighting the importance of pancreaticoduodenectomy in treating ZES (14–16). Most gastrinomas in patients with MEN1 are located in the duodenum rather than the pancreas (14). However, most pancreatic gastrinomas are sporadic (17). The distinction between duodenal gastrinomas/somatostatinomas and hyperplastic lesions is explained by the fact that a loss of heterozygosity (LOH) of *MEN1* and/or centromere 11 was demonstrated in approximately 50% of MEN1-associated duodenal NETs; however, hyperplasia or precursor lesions consistently lacked LOH on chromosome 11q13 (18). Further, gastrin microfoci in MEN1 have been described as being biologically aggressive gastrin-producing tumors (18), and it is still difficult to make a clear distinction. Moreover, duodenal NETs are typically considerably small, and endoscopic duodenal and gastric biopsy did not show submucosal tumors in Case 1, despite multiple attempts. Tumor metastases in locoregional lymph nodes are usually larger than the primary foci in the duodenum. This is called primary lymph node gastrinoma, in which only micro-gastrinomas <1.0 mm in diameter in the duodenum metastasize to the lymph node (19). Therefore, in patients with MEN1, screening for gastrinomas should focus on the proximal part of the duodenum, and sampling of other tissues, especially the pancreas, is not required (19).

### Patient prognosis and cause of death

In these cases, despite the fact that the patients suffered from multiple tumors and persistent ZES, the primary cause of death in Cases 1 and 3 was the presence of bronchial NETs G2. In Case 1, a bronchial NET G2 in the right lower pulmonary lobe was surgically resected when the patient was in his late 20s, without lymph node metastasis. A similar NET G2 tumor appeared in his right upper pulmonary lobe with multiple lymph node, rib, mediastinum, and liver metastases when he was in his early 50s, approximately 22 years after the first surgery. We were uncertain of whether the bronchial NET in his right upper pulmonary lobe was a metastasis of the first NET in his right lower pulmonary lobe or an independent multicentric NET due to the long time interval between the first surgery and appearance of the second NET.

The prevalence of bronchial NETs in patients with MEN1 is low (2%–8%), and multiplicity is relatively common (20). Determining whether a tumor is primary or metastatic in a ZES context can be challenging. In Case 1, the patient had multiple NETs in the bronchus, duodenum, pancreas, parathyroid gland, and pituitary gland, all of which had a similar histology with neuroendocrine features, especially those in the duodenum and pancreas. However, a metastatic NET may be differentiated from a primary one using biomarkers such as some peptide hormones produced by tumors, including serotonin, gastrin, insulin, glucagon, pancreatic polypeptide, somatostatin, parathormone, prolactin, and growth hormone, depending on the organ. In Case 1, bronchial NETs in his lower and upper lung lobes and mediastinal tumors were immunohistochemically positive for serotonin, with simultaneously elevated serotonin plasma levels. This confirmed that the mediastinal tumor cells were the same as those of the bronchial NETs in his right lower lobe. In addition, the mediastinal tumor was located in the posterior mediastinum and not in the anterior mediastinum, ruling out the possibility of it being a thymic NET. Cushing and carcinoid syndromes are the main neuroendocrine syndromes of bronchial ACs (21). Carcinoid syndrome is diagnosed based on the secretion of serotonin alongside histamine and bombesin (21). The prevalence of bronchial NETs among patients with MEN1 is 4.7–31.3%. Histopathological and radiological examinations, as well as patient outcomes, revealed a relatively indolent course and good prognosis (22). However, a cohort study carried out in France (the “Groupe d’étude des Tumeurs Endocrines” study) reported that of 1023 symptomatic patients with MEN1, bronchial NETs were identified in 51 of them (4.8%). Histological examination revealed 27 (53%) typical carcinoids (TCs), 16 (31%) ACs, 2 (4%) large cell neuroendocrine carcinomas, 3 (6%) small cell neuroendocrine carcinomas, and 3 (6%) AC-associated TCs. Moreover, nodal and distant metastases were observed in 37% and 16% of cases, respectively, in the cohort study (23). TC is a low-grade malignancy defined as NET G1. In contrast, AC is defined as NET G2, is more aggressive, and tends to metastasize to local lymph nodes, liver, bone, or brain (21). In the cases described, the proband and his father died of respiratory insufficiency caused by AC of the lungs and its metastases or multiplicity.

### Expression of menin in MEN1-associated tumors

Menin expression was observed in the nuclei of normal human tissue cells. Loss of menin expression as determined through IHC has been reported in MEN1-related tumors in retroperitoneal NETs (24), multifocal pancreatic NETs (25), adrenocortical carcinomas (26), and thymic NETs (27). However, these previous studies did not address the usefulness of menin IHC in MEN1 diagnosis. We observed that menin IHC patterns were inconsistent between cases; thus, its application in surgical diagnosis is yet to be elucidated.

In patients with MEN1, all somatic cells harbor a germline mutation of *MEN1*. A loss of heterozygosity (LOH) of *MEN1* and/or centromere 11 has been demonstrated in approximately 50% of MEN1-associated duodenal NETs (28). Allelic loss has been detected in tumors as small as 0.3 mm in diameter. As opposed to tumors, hyperplastic gastrin cells consistently lack LOH on chromosome 11q13 (28). In our study, IHC staining in duodenal gastrinomas varied from strong to weakly positive in micro-NETs of 0.7–8.0 mm in diameter. Two conceptualized issues exist in the pathological classification of proliferative lesions such as hyperplasias and neoplasias in endocrine organs. However, hyperplasias and neoplasias for small lesions, especially multiple nodules in an organ, are yet to be defined. Furthermore, there are no distinct morphological criteria to differentiate hyperplasias and neoplasias. Menin-negative IHC has been suggested as a possible indicator for MEN1-related tumors in previous studies (24–27, 29). Our results demonstrated that microadenomas in MEN1-related tumors exhibited a mixture of menin-positive and menin-negative cells.

**Figure 4:**
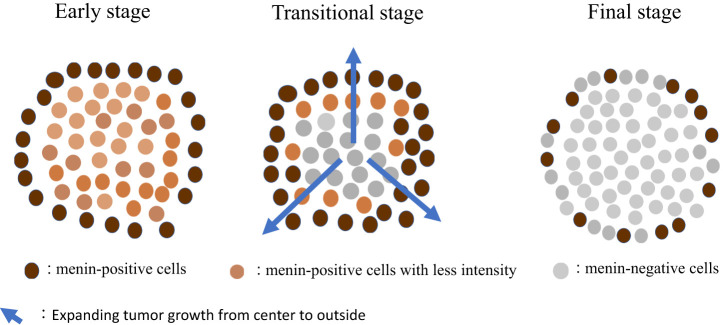
Suggestive hypothesis on the development of MEN1-related tumors. MEN1, multiple endocrine neoplasia type 1.

To avoid making subjective judgement, we used an imaging analyzer to observe menin IHC. Specimens were compared to positive and negative controls. The menin-positive to menin-negative cell ratio was different for each case i.e., 90% in normal pancreatic acini for the positive control, 35% in duodenal NETs, 12% in parathyroid adenomas, and 0.01% in adrenocortical adenomas. We hypothesized the relationship between tumorigenesis and loss of immunoreactive menin in pancreaticoduodenal small NETs in MEN1 as follows: “At the early stage of small NET development, tumor cells exhibit features of neuroendocrine cells, as indicated by the strong positivity for CgA, gastrin, or somatostatin, without loss of menin. During tumor growth, the menin-positive to menin-negative cell ratio becomes smaller i.e., approximately 90% with low menin intensity (early stage), 90–15% (transitional stage), and below 15% (final stage). Mature NETs are occupied by menin-negative cells despite the presence of residual menin-positive cells (up to 10%) at the periphery of the nodules (final stage)” (**Figure 4**). This hypothesis could explain the presence of varying menin IHC patterns, especially in pancreaticoduodenal small NETs in a context of ZES. This hypothesis is based on histological data and cannot be compared to the theory of the second hit of Knudson classically accepted for tumorigenesis due to tumor suppressor genes (30).

### Non-neuroendocrine tumors associated with MEN1

Menin IHC demonstrated positivity in the nuclei of thyroid follicular cells, with the lesion being compatible with nodular goiter but not follicular neoplasm. Lodewijk et al. (29) examined menin expression in non-tumorous lesions in patients with MEN1 and found that menin was expressed in all non-tumorous thyroid lesions. Furthermore, the prevalence of thyroid incidentalomas in patients with MEN1 was found to be the same as that in a matched reference group of patients without MEN1 (29). Thus, thyroid lesions are not considered MEN1-associated lesions. The adrenal myelolipoma demonstrated loss of menin in its adenomatous cortical component; this was not the case with the myelolipoma in Case 1. Previously reported cases of MEN1-associated adrenal myelolipoma did not exhibit LOH, suggesting that menin is not a contributing factor to myelolipoma (31).

In conclusion, pancreaticoduodenectomy is essential for ZES treatment, and bronchial NET is one of the important prognostic factors for patients with MEN1. Menin immunohistochemistry is not necessarily useful for diagnostic pathology on MEN1-related tumors especially for tumors of early stage due to the variability of immunostaining. MEN1 tumorigenesis was hypothesized based on menin IHC; however, further investigations are necessary.

## Data availability statement

The datasets presented in this study can be found in online repositories. The names of the repository/repositories and accession number(s) can be found in the article/supplementary material.

## Ethics statement

The studies involving humans were approved by Ethics Committee of the National Hospital Organization Hakodate National Hospital (approval number: R5-0315001). The studies were conducted in accordance with the local legislation and institutional requirements. The participants provided their written informed consent to participate in this study. Written informed consent was obtained from the individuals for the publication of any potentially identifiable images or data included in this article.

## Author contributions

Conceptualization and design of the work: NK and YH. Acquisition of the work: YH, NI, HK, ST, SY, SS, and MO. Gene analysis: SU. Formal analysis and interpretation of data: NK and HK. Drafting the manuscript: NK and ST. Final approval of the manuscript version to be published: YH, NI, HK, ST, SY, SS, SU, and MO. Equal contribution: These authors contributed equally to this work. All authors contributed to the article and approved the submitted version.
